# Troponin structure and function: a view of recent progress

**DOI:** 10.1007/s10974-019-09513-1

**Published:** 2019-04-27

**Authors:** Steven Marston, Juan Eiros Zamora

**Affiliations:** NHLI and Chemistry Departments, Imperial College London, W12 0NN London, UK

**Keywords:** Troponin, Thin filaments, Ca^2+^, Phosphorylation, Muscle regulation, Mutation

## Abstract

The molecular mechanism by which Ca^2+^ binding and phosphorylation regulate muscle contraction through Troponin is not yet fully understood. Revealing the differences between the relaxed and active structure of cTn, as well as the conformational changes that follow phosphorylation has remained a challenge for structural biologists over the years. Here we review the current understanding of how Ca^2+^, phosphorylation and disease-causing mutations affect the structure and dynamics of troponin to regulate the thin filament based on electron microscopy, X-ray diffraction, NMR and molecular dynamics methodologies.

## Introduction

**Fig. 1:**
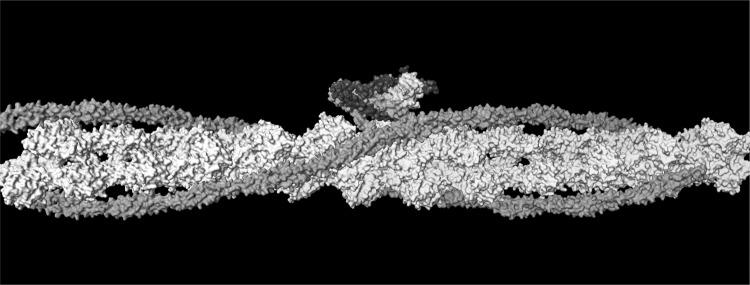
Model of the muscle thin filament based on the coordinates published by Pirani et al. (2006) showing the likely arrangement of actin (white), tropomyosin (red) and troponin (TnC pink, TnI magenta, TnT yellow), based on electron microscopy and X-ray diffraction. The barbed end (Z- band end) of the actin filament is on the left of the figure. (Color figure online)

The molecular mechanism by which Ca^2+^ binding and phosphorylation regulate muscle contraction through Troponin is not yet fully understood. Revealing the differences between the relaxed and active structure of cTn, as well as the conformational changes that follow phosphorylation has remained a challenge for structural biologists over the years (Fig. 1). Here we review the current understanding of how Ca^2+^, phosphorylation and mutations affect the structure and dynamics of troponin to regulate the thin filament.

**Fig. 2:**
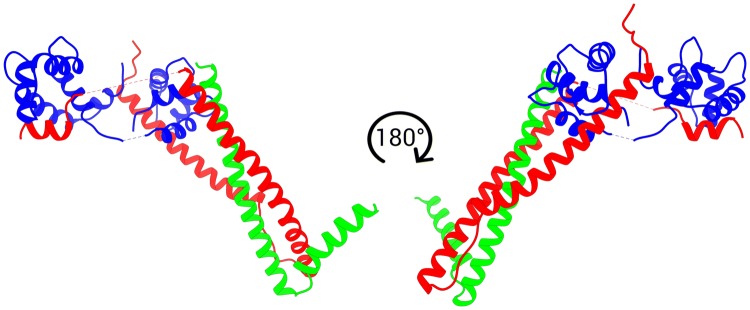
Front and back view of the 46 kDa core domain of human cardiac Troponin in the Ca^2+^-activated form. TnC is depicted in blue, TnT in green and TnI in red. PDB accession code 1J1D (Takeda et al. 2003). (Color figure online)

The most important achievement in troponin structure was the publication of a partial crystal structure of the core domain of human cardiac troponin (cTn) in the Ca^2+^ saturated state by Takeda et al. (2003) (Fig. 2). This structure reveals that in this state cTn adopts an L-shaped conformation, with different domains connected by flexible linkers. A rigid coiled-coil domain is observed between TnT and TnI and is referred to as the ‘IT arm’ in the literature. Additionally, the interaction between the N-terminal domain of cTnC and the C-terminal regions of cTnI (‘switch peptide’) is apparent and is usually referred to as the ‘regulatory head’. A later publication reported the activated state of chicken fast skeletal muscle troponin (sTn) in high resolution and a low-resolution structure of the inactive state (Vinogradova et al. 2005). These structures highlight the differences between the sTn and cTn isoforms. The cTnI isoform has an extended N-terminal region of 30 amino acids which is not present in the sTn isoform. This region was not present in the crystal structure of Takeda (2003) due to its disordered nature. Although the overall arrangement of the subunits of cardiac and skeletal muscle troponin is similar, the location of the regulatory head is different, with a smaller angle being formed between it and the IT arm in the sTn isoform. Additionally, in the Ca^2+^ form,the linker that joins the two domains of TnC is helical in the sTn molecule, while in cTn it is disordered. Finally, the Ca^2+^-free structure of sTn indicates dissociation of the switch peptide of cTnI from the hydrophobic cleft that is bound to in the Ca^2+^-saturated conformation.

The cTn crystal structure presents the orientation between the IT arm (*α*-helical coiled-coil structure formed from part of cTnI and cTnT) and the regulatory head (both terminal regions of cTnI and the N-terminal domain of troponin C (NcTnC). Further nuclear magnetic resonance (NMR) studies focused on revealing the sections of the complex that are missing from Takeda’s structure, such as the cardiac-specific NcTnI region, the cTnI inhibitory region, the C-terminal regions of both cTnI and cTnT and all of the N terminal domain of TnT (over 200 residues). An NMR structure of the N terminus of troponin I (NcTnI) region published by Howarth (2007), both in the unphosphorylated and bisphosphorylated forms, showed substantial conformational differences between the two. Recent NMR studies have described this peptide as an intrinsically disordered region (IDR) in the non-phosphorylated form. Its interaction with NcTnC also seemed to position it relative to the IT arm, and possibly modulate its interaction with the cTnI switch peptide. There are conformational changes after phosphorylation of NcTnI that appear to disrupt this interaction (Hwang et al. 2014).

**Fig. 3:**
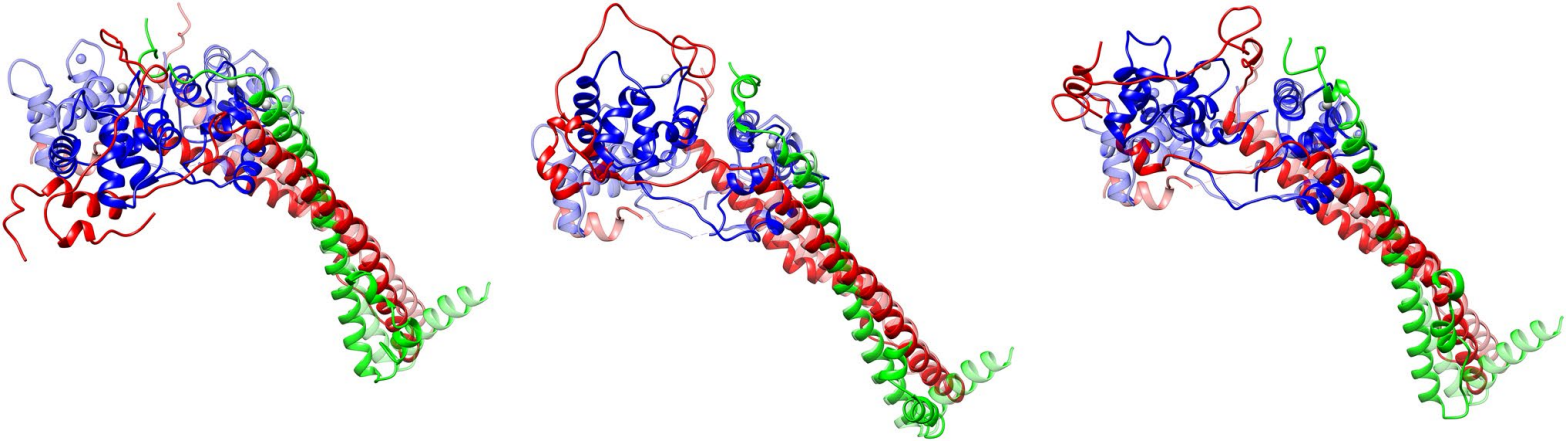
The three most populated structures of human cardiac troponin determined by molecular dynamics simulations overlaid over the Takeda et al. structure of hcTn (pale structure) TnC is depicted in blue, TnT in green and TnI in red (Zamora 2019). (Color figure online)

Recently the structure of the cardiac troponin core has been investigated by molecular dynamics simulations (Cheng et al. 2014; Papadaki and Marston 2016; Zamora et al. 2016); (Fig. 3).

These studies confirm the structure of troponin derived from X-ray crystallography for the rigid parts of the molecule and provide information about the structure and dynamics of the intrinsically disordered segments. The regulatory head and the IT arm are found to be relatively rigid and similar to the crystal structure but the relative disposition of the two domains is extremely variable. The structure of troponin cannot be realistically represented by any static model, but, for comparison with the X-ray model, we show the most populated structures of the three most probable macrostates of cardiac troponin with Ca^2+^ bound derived from MD calculations (Zamora 2019; Zamora et al. 2016) (Fig. 3).

The location of NcTnI near NcTnC, the hinge motion of the linker peptide region between the regulatory head and the C terminus/IT arm and the potential interactions of the C-terminus of troponin T with the TnI and TnC in the hinge region is evident from these structures.

## Troponin C

**Fig. 4:**
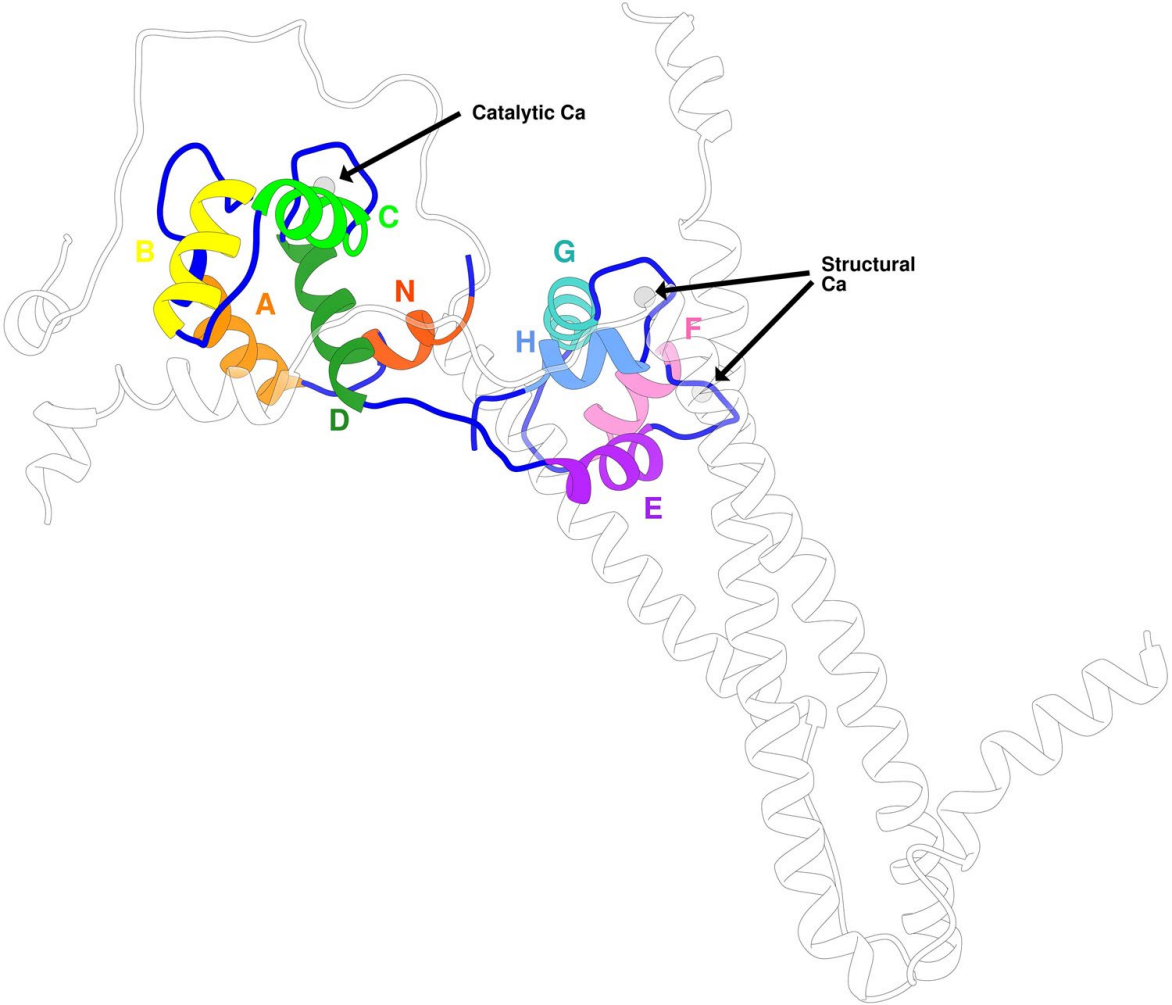
The cTnC molecule with its nine helices highlighted. Subunits cTnI and cTnT are transparent for clarity. The troponin structure is the original Takeda structure with the disordered segments of TnI and TnT modelled (Zamora et al. 2016)

**Fig. 5:**
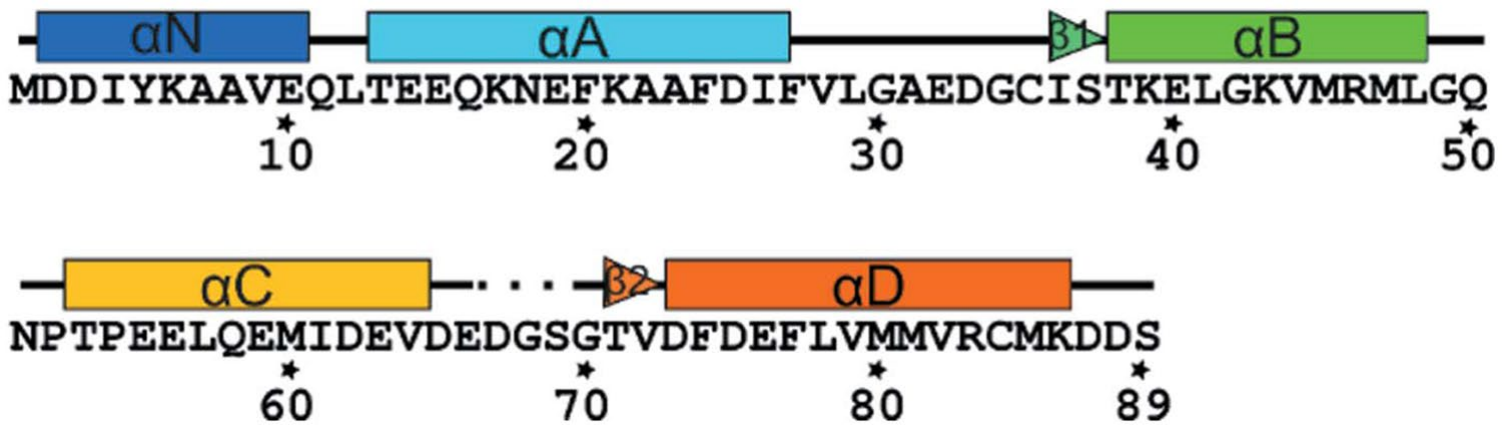
Sequence and secondary structure of cTnC N-terminal domain. Numbering is for human cardiac troponin C (P63316)

The troponin C (TnC) molecule owes its name to the fact that it is the Ca^2+^ sensing subunit of the Tn complex. In the human heart, it is expressed as a 161-residue long protein made up of two globular domains joined by a flexible hydrophobic linker. It has a molecular weight of around 18 kDa. TnC contains nine short *α* helices named in alphabetical order except for the first, helix N (Figs. 4 and 5). In total, TnC contains four structural motifs known as EF-hands capable of binding positive divalent cations. Each EF-hand consists of two *α*-helices interconnected by a loop with negatively charged residues that coordinate the positive ion. EF hand I (residues 16 to 51) and EF-hand II (residues 52 to 87) have a low affinity for Ca^2+^ (*K*_*a*_≈ 10^5^ M^−1^) but are highly selective to it. These different behaviours have resulted in the naming of the Ca^2+^ ions that bind to site I and II as the ‘catalytic’ or ‘regulatory’ Ca^2+^, while sites III (residues 92 to 127) and IV (residues 128 to 161) are known as’structural’ Ca/Mg sites; these are always occupied under physiological conditions (*Ka *≈ 10^7^ M^−1^), and other divalent cations such as Mg^2+^ and Cd^2+^ can also bind to these sites. In the cardiac isoform, the first EF-hand does not bind Ca^2+^ at any concentration thus site II is alone responsible for Ca^2+^sensing during muscle contraction (Figs. 4, 5, Table 1).

**Table 1:**
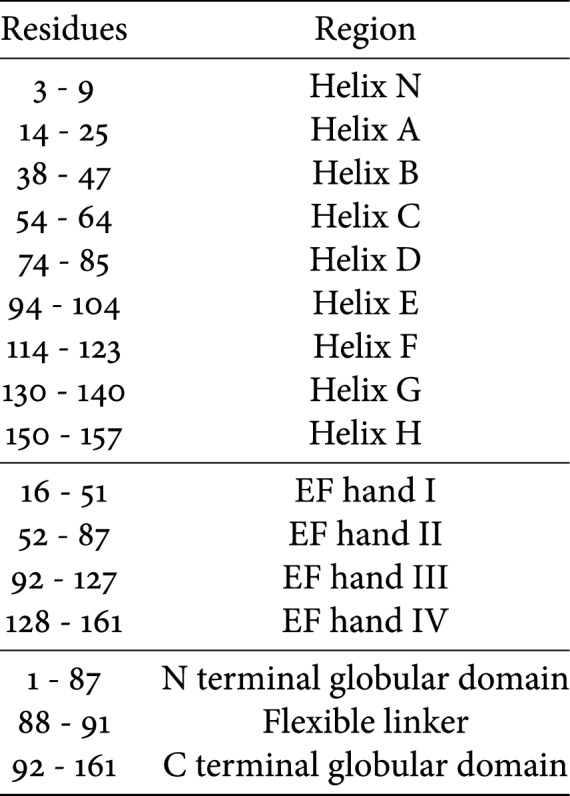
Standard nomenclature and numbering for the structural elements of human cardiac TnC (P63316)

### N-terminal domain of TnC

**Fig. 6:**
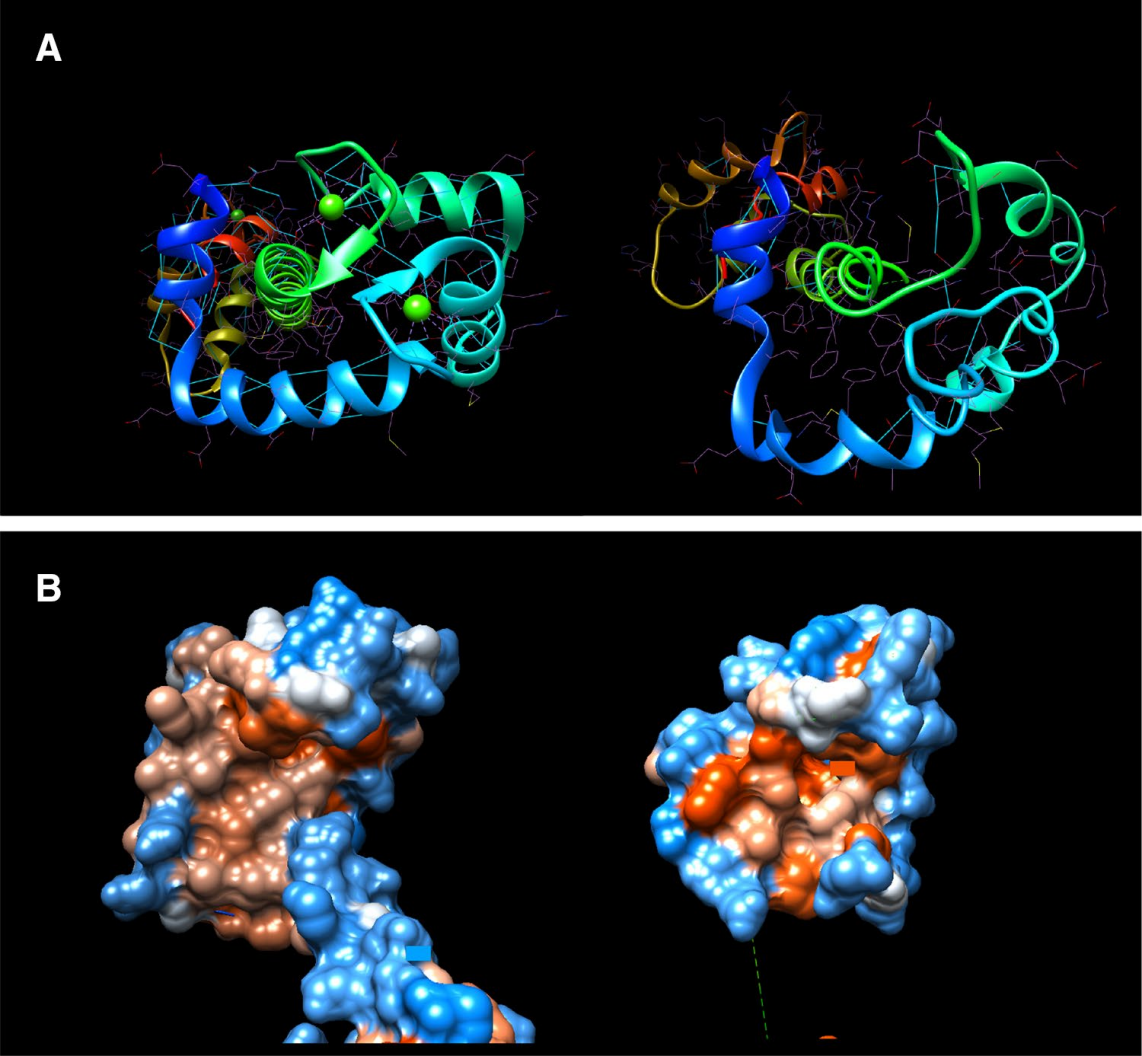
Ca^2+^ dependent changes in the structure of the N-terminal lobe of skeletal troponin C. These images are from the X-ray structure of whole skeletal muscle troponin (1ytz and 1yv0) (Vinogradova et al. 2005) with TnI and TnT edited out for clarity. Thin blue lines are putative hydrogen bonds. Thin red lines are the residues of the hydrophobic patch. **a** Ribbon diagram of (left) the Ca^2+^-saturated sTnC and (right) Ca^2+^-free TnC, viewed from above the short beta sheet formed by amino acids just preceding helices B and D. This structure is disrupted, the two EF hands (AB and CD) move away from each other and the angle between helix A and B changes from 135° to 81° in the absence of Ca^2+^. **b** Hydrophobicity surface rendering of sTnC, viewed from the underneath. Maximum hydrophobicity is brown and minimum is blue. In the Ca^2+^ bound state an extensive hydrophobic surface is presented to the TnI switch peptide (see Fig. 8) (left) that is closed off in the absence of Ca^2+^ (right). (Color figure online)

In its role as Ca^2+^ sensor, troponin C undergoes an extensive concerted structural rearrangement when Ca^2+^ binds from the so-called ‘closed’ state to an ‘open’ state. The two EF hands move closer to each other with the formation of a short hydrogen-bonded beta-sheet motif between them and reorientation of the helices resulting in the opening of a hydrophobic patch that creates a binding site for the switch peptide of troponin I (Figs. 5, 6).

## Troponin I

**Fig. 7:**
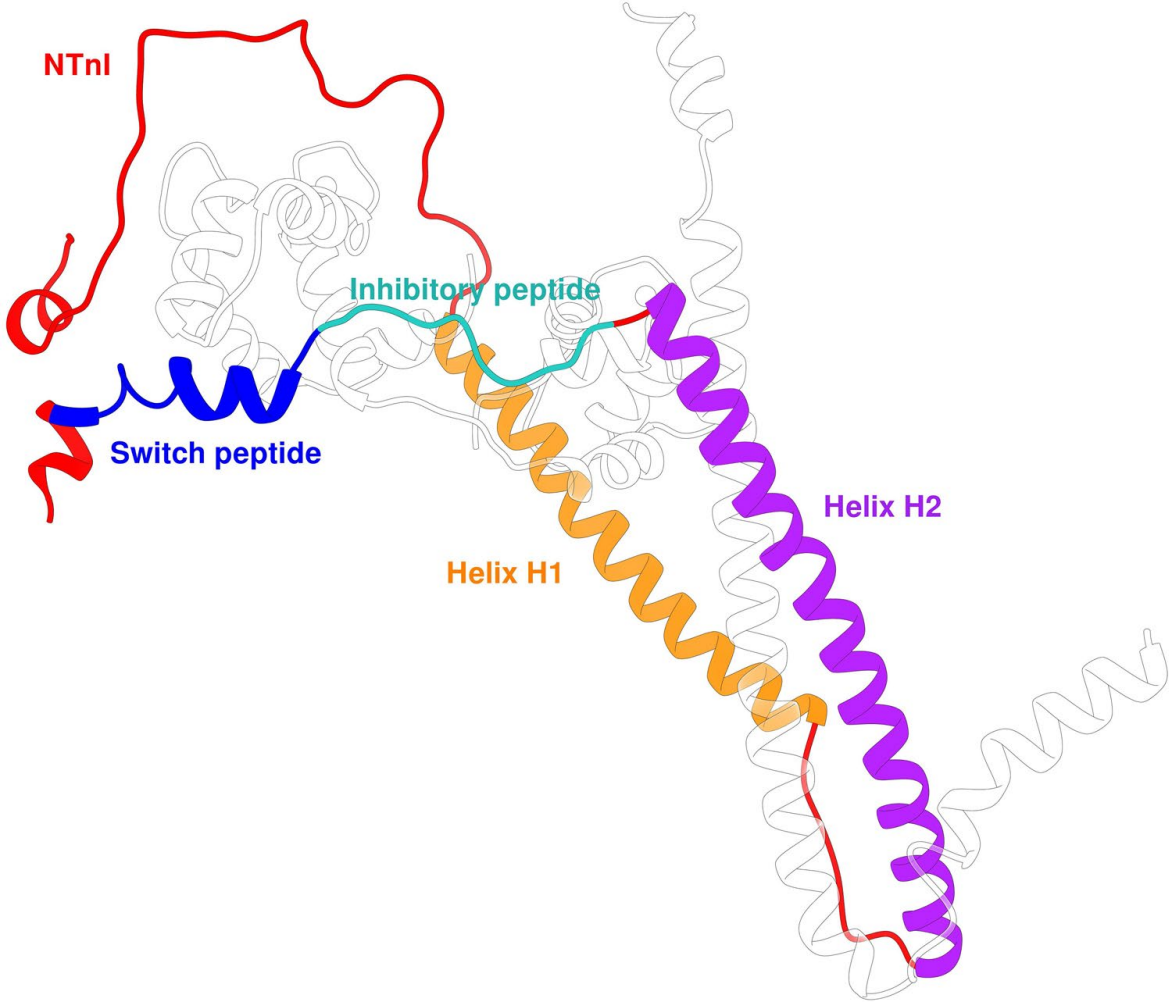
The cTnI molecule with its relevant structural regions highlighted. Subunits cTnC and cTnT are transparent for clarity. The switch peptide corresponds to Helix H3. Residues 172 to 210 of cTnI are not present in this structure (Zamora et al. 2016)

Troponin I (TnI) is expressed in three different isoforms: cardiac, slow skeletal and fast skeletal muscle TnI. During embryonic development, slow skeletal TnI is expressed in the cardiac tissue, but it is replaced by cardiac TnI (cTnI) after birth (Marston and Redwood 2003), with the expression of this isoform being exclusive to the cardiac tissue. The mature form of human cTnI consists of a 210-residue long protein (209 after the first Met residue is removed) with a molecular mass of approximately 24 kDa. This protein is arranged as four *α* helices intercalated by flexible disordered regions. Structurally, it can be classified into the following domains: the cardiac-exclusive N terminal domain (NcTnI), the structurally rigid IT arm, the “inhibitory” peptide, the switch peptide and the C-terminal domain (Fig. 7 and Table 2).

**Table 2:**
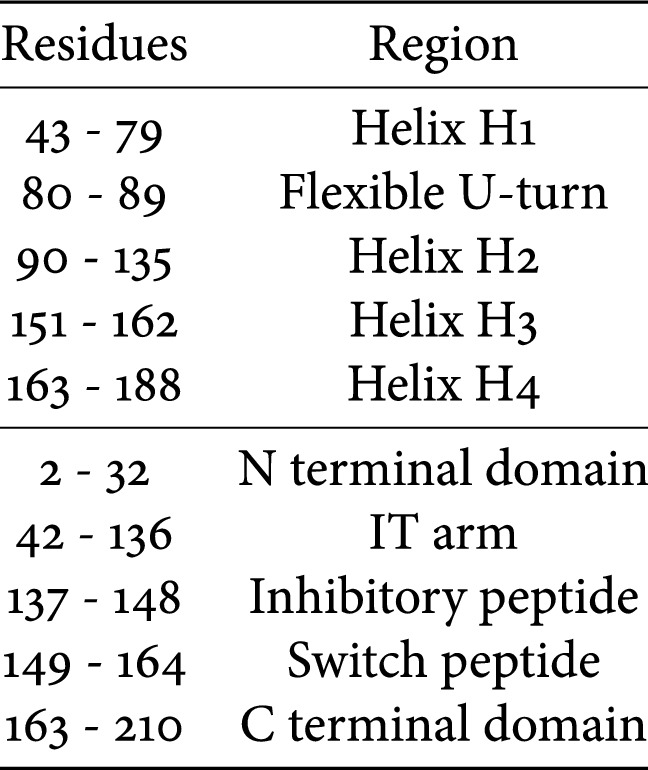
Standard nomenclature and sequence numbering for the different structural regions of human cardiac troponin I (P19429)

The NcTnI region is exclusively expressed in the cardiac isoform of TnI. It consists of an acidic region (residues 2 to 11) and a Xaa-Pro motif (residues 12 to 18) that can form a short and unstable *α* helix. In this region two adjacent Serine residues are located (Ser 22 and Ser 23) which are the targets of PKA-mediated phosphorylation. A structure has been proposed for this peptide in isolation, however in the whole troponin molecule it is disordered, although its location over the N-terminal domain of TnC has been predicted from modelling and molecular dynamics simulations (Cheng et al. 2014; Howarth et al. 2007; Hwang et al. 2014; Zamora et al. 2016) (Figs. 3, 7).

The IT arm is a rigid coiled-coil of *α* helices of cTnI and cTnT. It is formed by the directionally-opposed *α* helices H1 and H2 (residues 43 to 79 and 90 to 135, respectively). These two helices are connected by a flexible U-turn (residues 80 to 89). The function of the IT arm is structural, as it is the least mobile region of the Tn complex. It serves as an anchoring region for the C-terminal domain of troponin C.

Upstream of helix H2 is the so-called ‘inhibitory peptide’ of cTnI (residues 137 to 148), which contains six positively charged residues. This short region owes its name to the observation that the peptide by itself can interact with actin-tropomyosin and inhibit actomyosin ATPase at a substoichiometric ratio to actin (Van Eyk et al. 1997). The name is, however, misleading: when incorporated into the whole troponin it is a flexible linker between the TnC N terminal domain and the TnC C-terminus/IT arm domain and is not directly involved in inhibition. The existing structural studies on this region have provided conflicting views on whether this region has any defined structure or not.

**Fig. 8:**
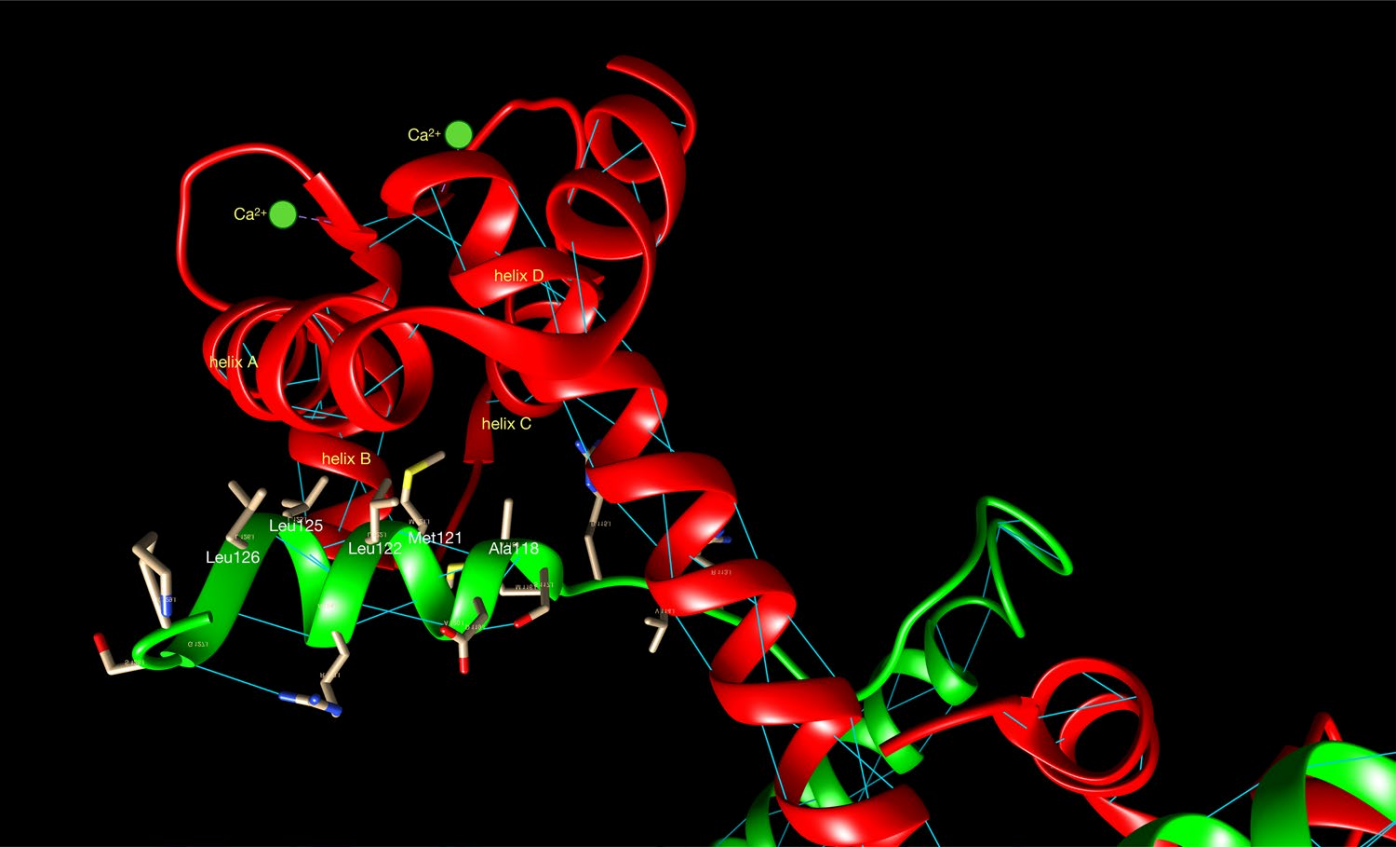
The binding of the skeletal muscle TnI switch peptide, helix3 (green), to the hydrophobic patch of skTnC- Ca^2+^ (red). The switch peptide is an amphipathic helix with hydrophobic residue on the face interacting with TnC (Ala118, Met121, Leu122, Leu125, Leu126). Rendered from Vinogradova’s X-ray structure 1ytz with segments of TnI C-terminal to Helix 3 and TnT are removed for clarity. (Color figure online)

The switch peptide of cTnI includes helix H3 (residues 151 to 162) which at high Ca^2+^ concentration interacts with the N terminal region of cTnC through the hydrophobic patch that is otherwise closed (Fig. 6b). The binding of TnI to TnC- Ca^2+^ is the key interaction of muscle activation since it re-orientates the extreme C-terminal domain of TnI so that it is not capable of interacting with actin and tropomyosin (Fig. 8).

The final domain of cTnI is the C-terminal region, spanning residues 163 to 210, which interacts with actin at low Ca^2+^ concentrations. The structure of this domain is not fully understood since it was not resolved in any of the X-ray diffraction structures.

**Fig. 9:**
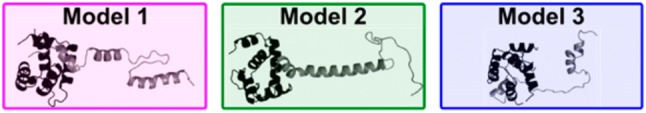
Three models of the structure of the TnI C-terminal domain with the preceding switch peptide docked on TnC Ca^2+^ (left). Model 1 is (Murakami et al. 2005) (NMR, NOE), model 2 is (Blumenschein et al. 2006) (NMR, CSI) and model 3 is (Takeda et al. 2003). From (Metskas and Rhoades 2015), with permission

A number of NMR studies have addressed the structure of this domain with conflicting results (Fig. 9). However, a recent study using single-molecule Förster resonance energy transfer and computational modelling has produced a coherent description (Metskas and Rhoades 2015). In this analysis none of the models is favoured, instead, TnI C terminus is viewed as intrinsically disordered and can exist in many low energy conformers that are kinetically connected through an ensemble of disordered transition states. In general, the conformers are extended and it has been suggested that TnI C-terminal inhibitory binding to actin could be described by a “fly-casting mechanism” that would allow TnI C terminus to explore the surface of actin at a distance of 5–7 nm from the troponin core. Upon finding its contact site it would form a folded complex on actin, thus ‘‘reeling in’’ the whole troponin complex to the position that it occupies once TnI is bound to actin (Blumenschein et al. 2006; Shoemaker et al. 2000).

## Troponin T

The troponin T (TnT) subunit is the largest of the three and mainly has a structural role. It is responsible for the fixation of the complex to the thin filament surface and the positioning of the core domain of cTn relative to the direction of the actin filament. Similarly to cTnI, there exist several isoforms of this protein which are differentially expressed in the heart, the fast and slow skeletal muscle, as well as different isoforms expressed during embryonic development. Each tissue-specific gene can express a variety of protein isoforms by alternative splicing. The description here is for the dominant adult cardiac muscle TnT isoform (TNNT2 gene isoform 6 (P45379-6), commonly referred to as T3 (Anderson et al. 1991; Bayliss et al. 2012).

The mature form in the adult human heart is a 288-residue long protein (287 after the first Met residue is removed) with an approximate molecular weight of 36 kDa. Despite its structural role, the majority of the cTnT molecule has not been resolved experimentally, and most of what is known about has been gathered from studies of partial structures.

**Fig. 10:**
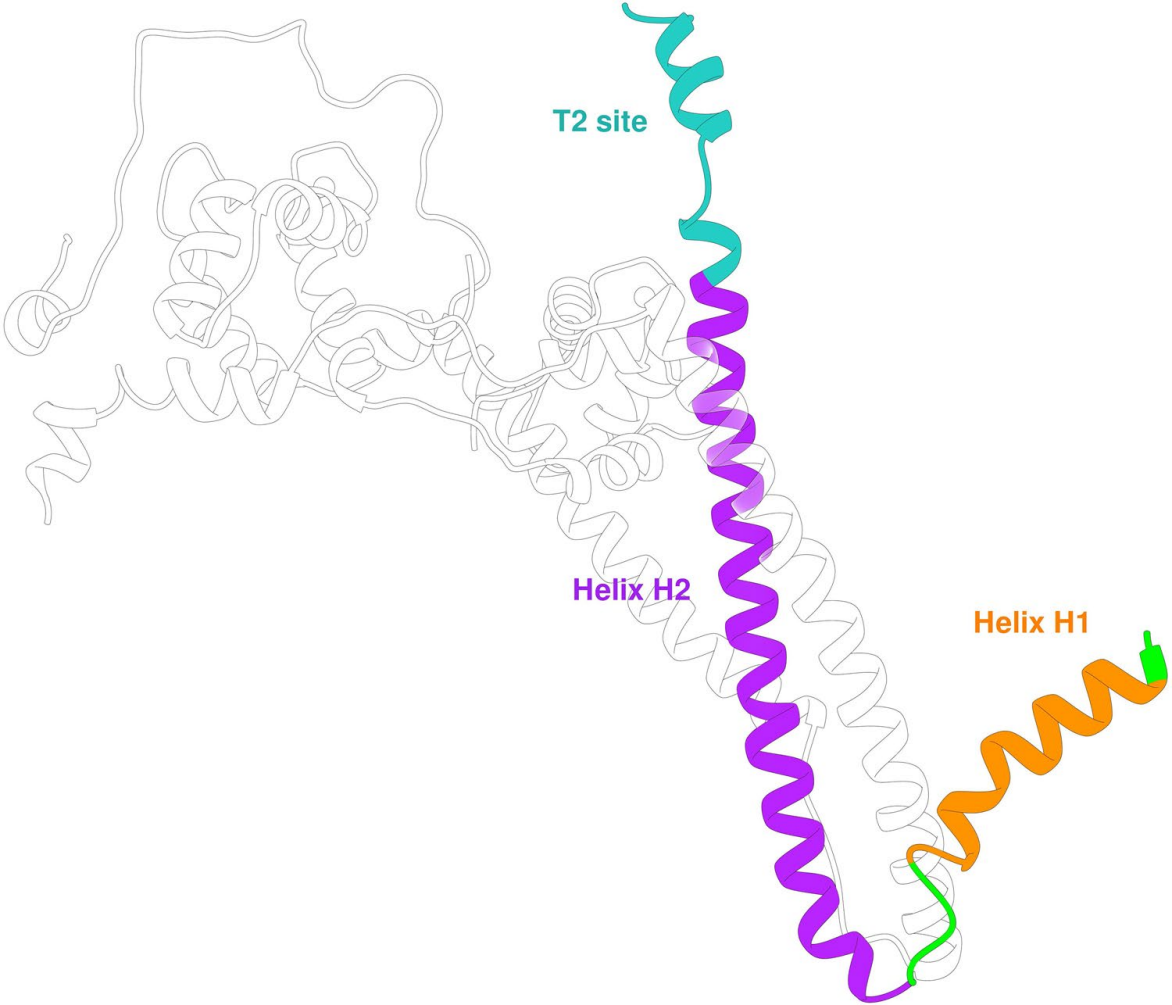
The cTnT2 domain with its relevant structural regions highlighted. Subunits cTnC and cTnI are transparent for clarity. Residues 1 to 201 of cTnT are not present in this structure

The Troponin T amino acid sequence is divided into three structural domains: The N terminal domain, the linking domain and the C terminal domain (Table 3). The C terminal domain is part of the core troponin complex whilst the rest is associated with tropomyosin. The N-terminal segment, named TnT1, has an extended structure that includes regions of single alpha-helix that bind to tropomyosin. The X-ray diffraction study of (Cabral-Lilly et al. 1997) established the position and orientation of TnT1 fragment binding in parallel with tropomyosin. It is likely that TnT1 plays a part in stabilising the overlap zone between two tropomyosin molecules (Murakami et al. 2008; Palm et al. 2001). Functionally, the presence of TnT1 in the thin filament independently enhances the cooperativity of the Ca^2+^ switch, presumably via its interactions with tropomyosin (Schaertl et al. 1995) (Fig. 10).

**Table 3:**
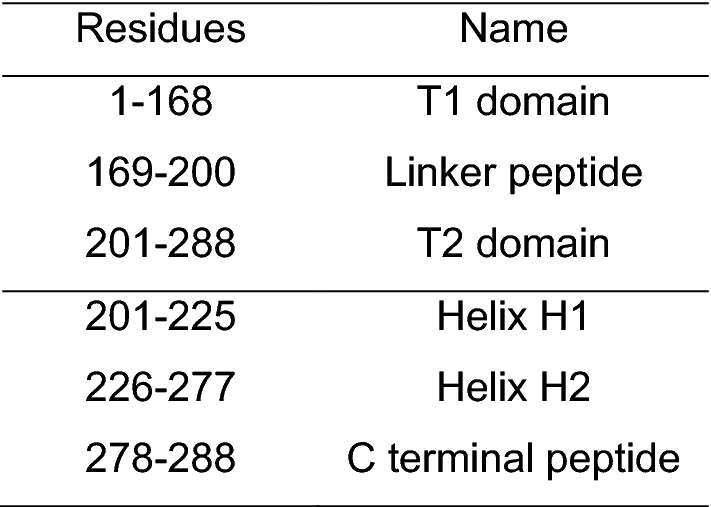
The domains of troponin T, standard numbering for cTnT isoform 6 (P45379-6)

**Fig. 11:**
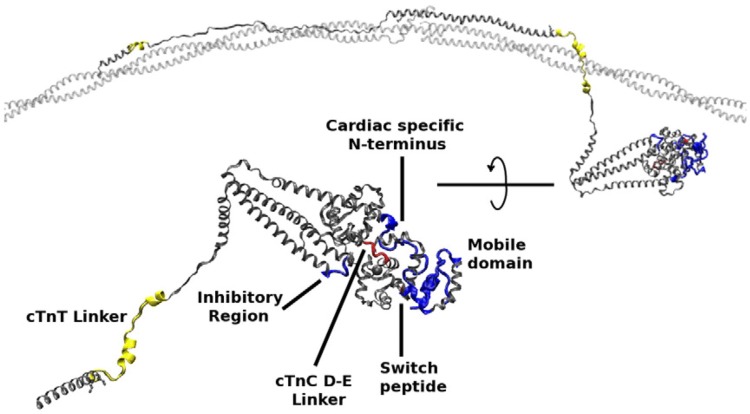
Model of troponin T and its interaction with tropomyosin and troponin I. WT average structure (grey) with regions that are structurally sensitive to TNT1 mutations highlighted. The highlighted regions are colored as a function of their subunit: yellow, cTnT; blue, cTnI; and red, cTnC. From (Manning et al. 2012) with permission. (Color figure online)

There is a flexible linker between TnT1 and TnT2. This is essential for locating and anchoring the troponin complex onto the actin -tropomyosin filament independent of [Ca^2+^]. It appears to be largely disordered, however, Helix H1 of TnT in the crystal structure may be considered as the C-terminal end of this domain. Both the tropomyosin overlap zone and the linker peptide with Helix H1 are hot spots for mutations that cause cardiomyopathies, indicating the role of these regions in modulating the troponin switch mechanism despite being outside the troponin core. The possible structure of TnT1 and the link peptide interacting with tropomyosin has been largely determined by modelling studies, for example, Fig. 11 and, for now, remains hypothetical. (Manning et al. 2012; Orzechowski et al. 2015).

**Fig. 12:**
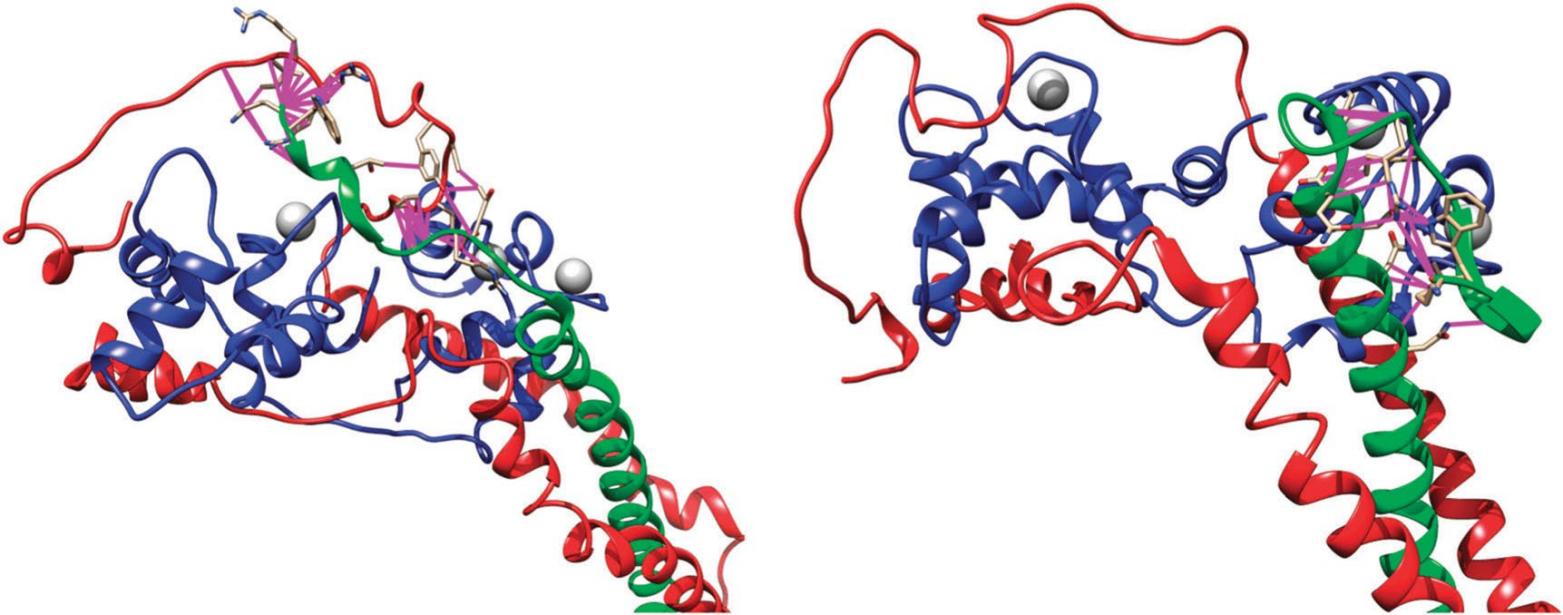
The two most probable conformations of the C-terminus of troponin T determined by molecular dynamics simulations (Zamora et al. 2016). Left is the more common configuration. TnT is in green, magenta bars represent hydrogen bonding between TnT and TnI or TnC. (Color figure online)

The TnT2 domain is upstream of the linker region and forms an integral part of the troponin core (residues 226 to 271) (Fig. 10). TnT Helix H2 is the central component of the IT arm where it forms an antiparallel coiled-coil with TnI Helix H2 that spans the length of troponin and also interacts with TnI helix H1. At the end of TnT helix H2, there are interactions with the C terminal lobe of cTnC. The last sixteen residues of CcTnT were not resolved in the structure of Takeda et al. (2003), indicating their flexibility. They have functional relevance to troponin since an HCM mutation leading to truncation of the last 14 amino acids is a well-known cause of hypertrophic cardiomyopathy (Franklin et al. 2012; Messer et al. 2016; Thierfelder et al. 1994). In their complete model of the human cardiac muscle troponin core, Zamora et al. used molecular dynamics to assess its conformation and found that the C terminal 16 amino acids are commonly unstructured and can interact with the TnI ‘inhibitory’ and N-terminal peptides in the hinge region of troponin or, less frequently, with TnT Helix 2 and C-terminal domain of TnC (Zamora et al. 2016) (Fig. 12).

## Troponin in the thin filament

Whilst the isolated troponin molecule is a suitable subject for detailed structural analysis, in vivo troponin is a fully integrated component of the thin filament along with tropomyosin and actin. Troponin’s interactions with actin and tropomyosin are the basis of Ca^2+^-dependent regulation of the thin filament (Gordon et al. 1997) and all the measurements of troponin regulatory function involve the whole thin filament interacting with myosin. The fundamental structure of the thin filament is the double helix of actin monomers (13 per full turn). The elongated tropomyosin molecule follows the actin helix at a ratio of 1 Tm/7 actin and the tropomyosin molecules are joined end-to-end producing a continuous strand. One troponin is located on the thin filament per tropomyosin (i.e. per 7 actins). Troponin-tropomyosin in the two strands of actin helix are orientated in parallel with their ends in register. The interactions between actin, tropomyosin and troponin in one strand of the helix are fundamental but additional interactions may be possible across the filament, since two troponins are bound at nearly the same level.

**Fig. 13:**
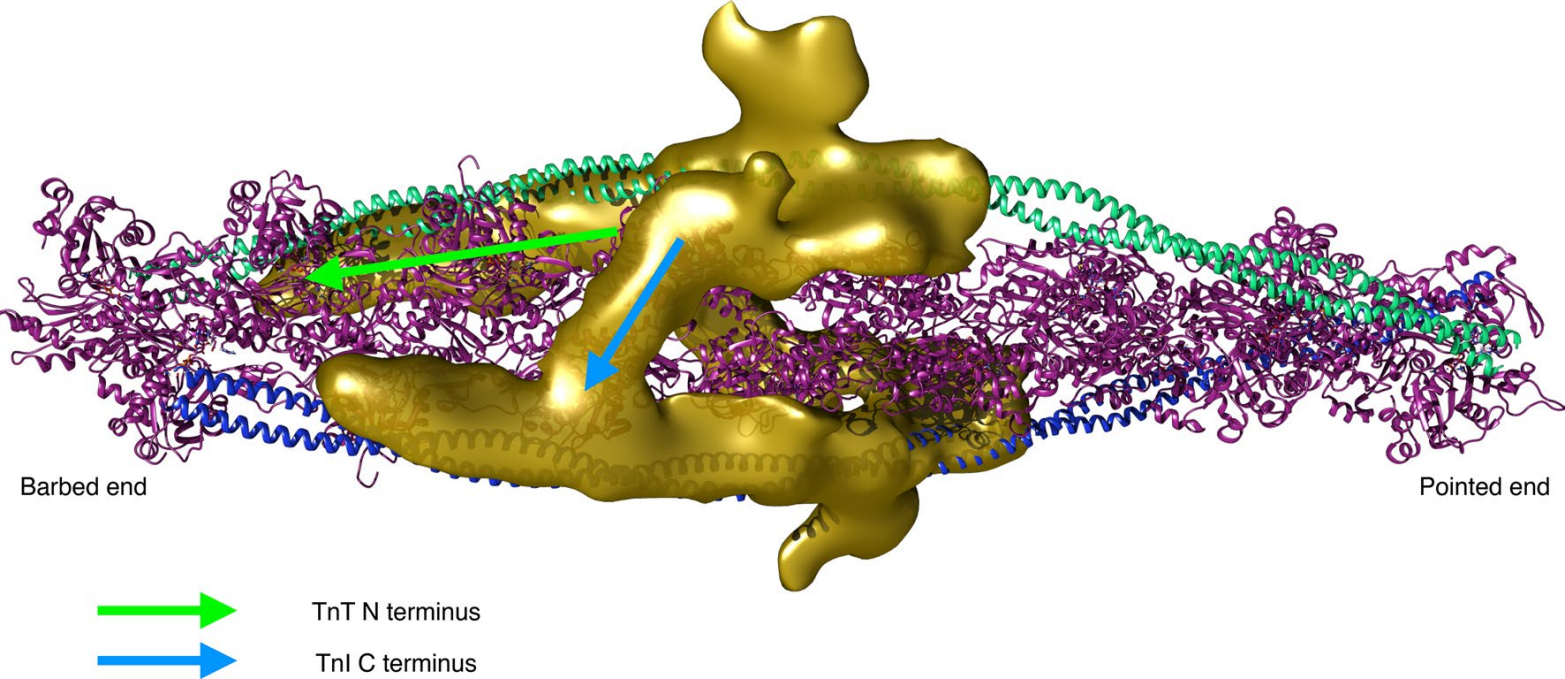
Troponin rendering onto the thin filament. The thin filament is represented by two coiled-coil tropomyosin monomers shown in cyan and blue over a core of actin monomers shown in ribbon view in magenta. The calculated troponin electron density is represented in gold. The image data was kindly supplied by Dr William Lehman (Boston University, MA, USA) based on Yang et al. (2014). Image modified from Papadaki and Marston (2016) with permission

Thin filament structure has mainly been studied by electron microscopy and fibre X-ray diffraction (Lehman 2016). A recent model of troponin orientation and position in the thin filament in the absence of Ca^2+^ has been achieved using electron microscopy by Yang et al. (2014). This combines the electron density envelope from electron microscopy, fibre X-ray diffraction (Poole et al. 2006) and X-ray crystallography structures of the component proteins with routines for fitting the structures into the electron density envelope. For actin and tropomyosin in the active and relaxed states the structures are well established whereas for troponin the fitting is still controversial and the low resolution allows for multiple possible fits. In Yang et al’s interpretation, the longitudinal element parallel with tropomyosin would be the T1 domain of troponin T. One of the more striking elements of these reconstructions is the ‘bar’ reaching across the filament (Fig. 13). The reconstruction is in the absence of Ca^2+^ and so likely shows how the C terminus of TnI reaches across actin and binds near tropomyosin in the blocked state.

**Fig. 14:**
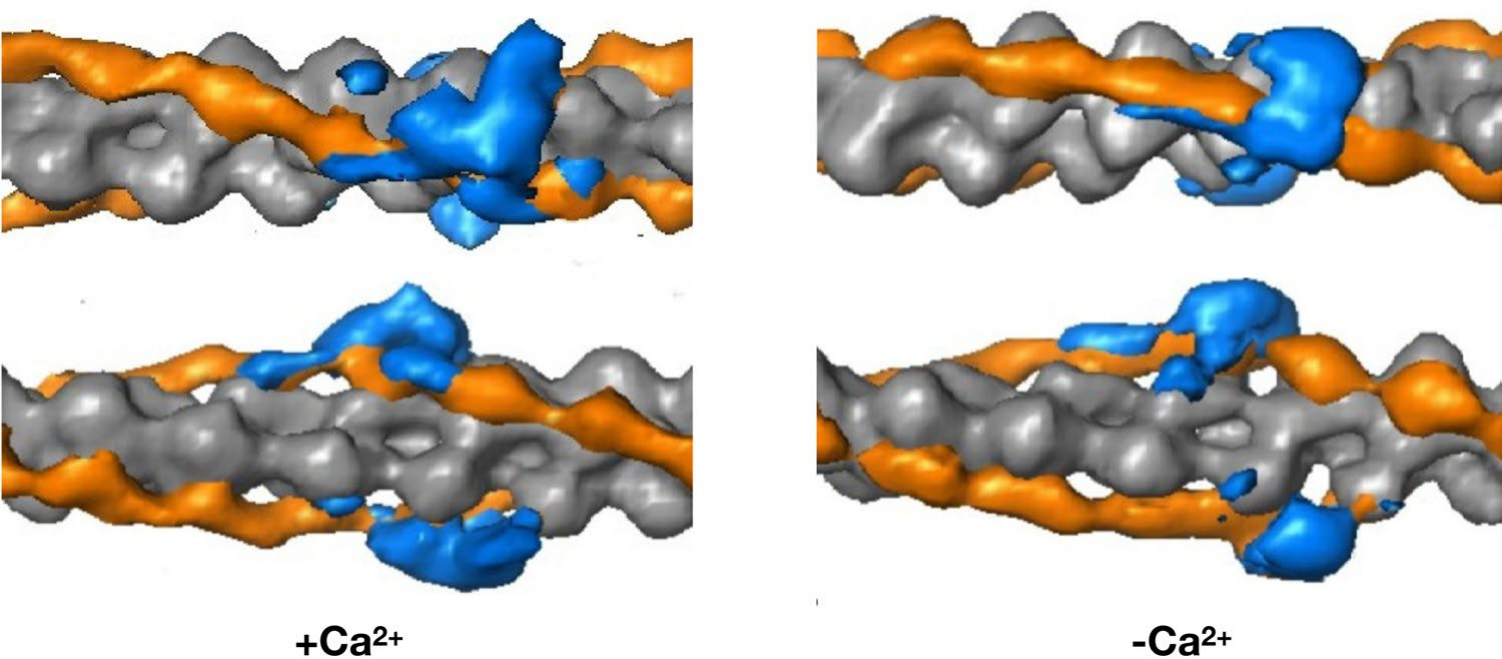
The barbed end (Z- band end) of the actin filament is on the left of the figure. Difference density maps calculated by subtracting docked F-actin (grey) and tropomyosin (orange) models from the single particle reconstructions leaving density attributable only to troponin (blue). Filament shown in two orientations rotated by 90°. The lower orientation approximates to the orientation in Figs. 1, 13 and 16. From (Paul et al. 2017) with permission. (Color figure online)

Another model, produced by Paul et al. is proposed to be more correct since it uses a non model-based single particle analysis of the electron micrographs (see Fig. 1). The main features are the same but, interestingly, this model shows significant differences in the location of the putative Tn mass on the thin filament in the presence and absence of Ca^2+^ and also fits the troponin core X-ray structure into the electron density envelope differently (Paul et al. 2017) (Fig. 14).

**Fig. 15:**
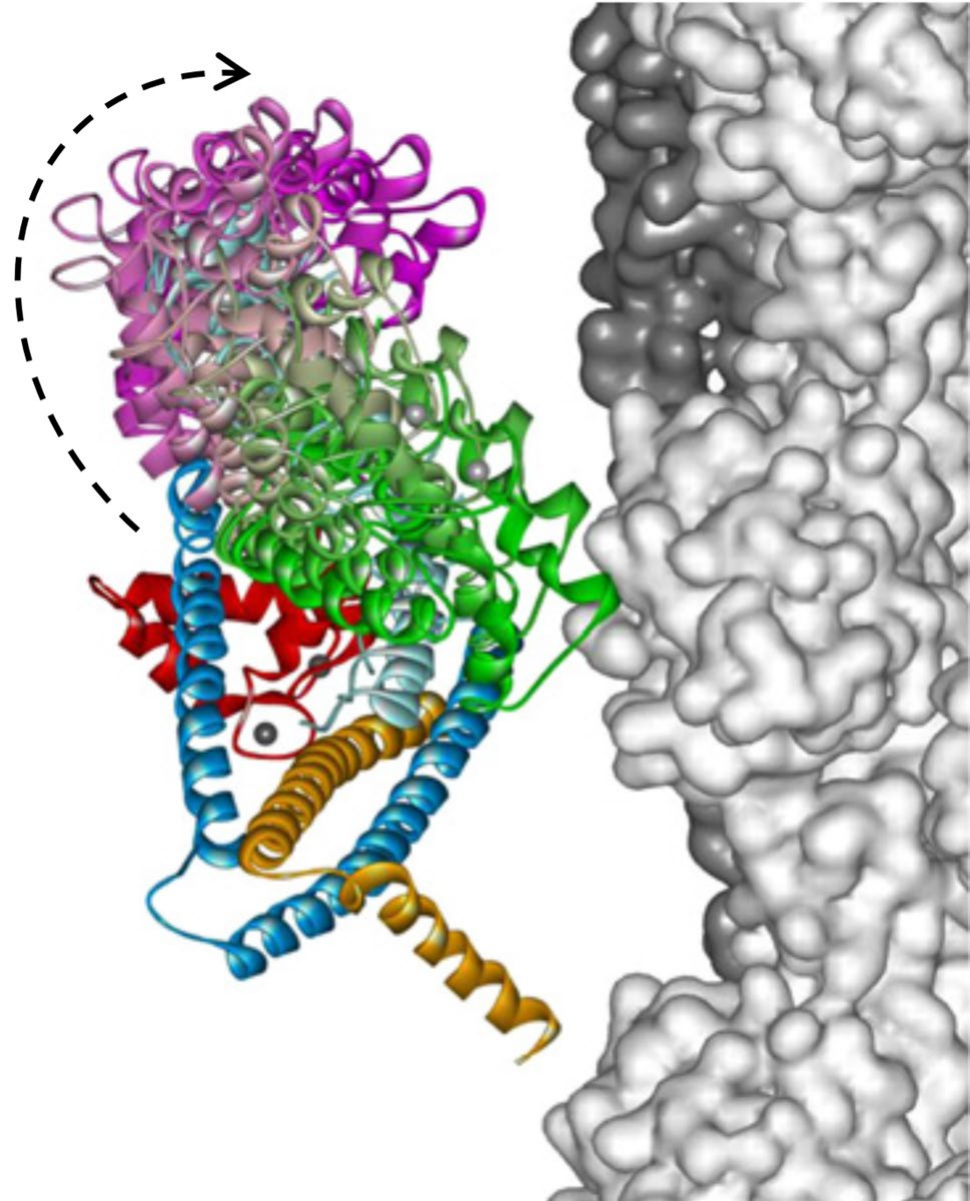
Troponin orientation on actin determined from fluorescence polarization measurements. The three metastable positions of the N-terminal domain are superimposed: green, A1, light purple, A2 and magenta, A3. The arrow indicates the transition from relaxed to active (+Ca^2+^) contraction (Sevrieva et al. 2014) with permission. (Color figure online)

All electron microscopy-based studies suffer from the problem that they only visualize a static state of the thin filament and that the visualization procedures (negative stain or cryopreservation) may change the thin filament structure. Since the thin filament is a dynamic system, methodology that can measure dynamics are needed. Recently, Sun et al. have used fluorescence polarization of probes fixed on troponin to directly determine the orientation of the Tn complex and the effect of Ca^2+^ within a contracting muscle (Knowles et al. 2012; Sevrieva et al. 2014). The overall orientation of troponin agrees with the Yang model, although the orientation of the IT is more slewed across the filament. Importantly, the IT arm barely changes in the presence of Ca^2+^, whilst the TnC N-terminal domain orientation does. NTnC is located close to the actin filament and yet it is capable of substantial hinge movement: three interconverting metastable conformations were calculated in the steady state. Figure 15 shows the three conformations superimposed with A1 predominating in the absence of Ca^2+^ and A3 being favoured when Ca^2+^ is bound.

**Fig. 16:**
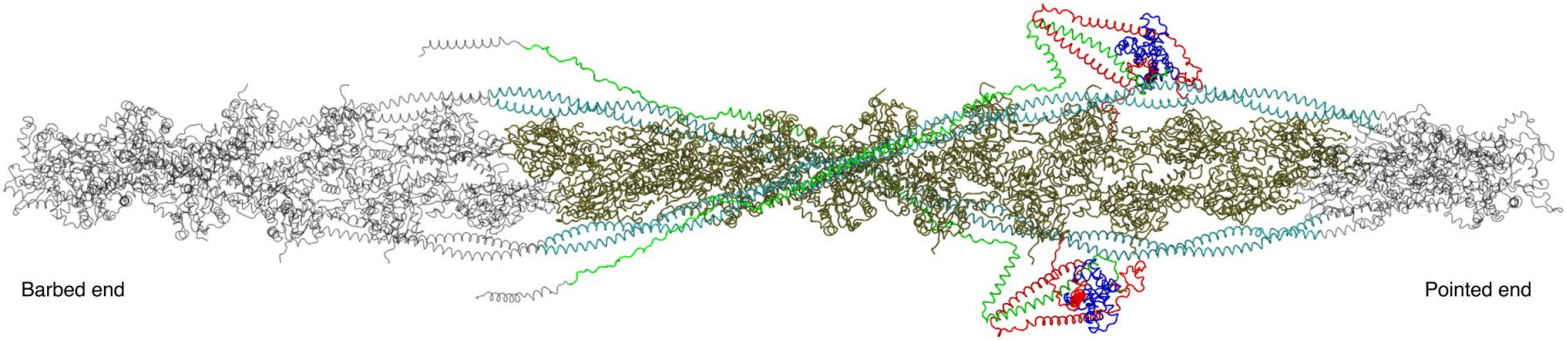
Model of the thin filament built by Williams et al. based on computational chemistry. (Williams et al. 2016) in grey. The shorter model of Gould and Zamora is coloured. (Color figure online)

An alternative approach is through computational chemistry. Full atomistic simulation of the complete thin filament structure has been achieved by Schwarz (Williams et al. 2016) through the positioning of troponin on the actin filament using docking calculations. The model of the thin filament is based on individual protein structures and known relationships, as described above, and then refined to avoid atom–atom clashes followed by iterative calculations of a best structure by energy minimization. Such a model may then be developed in more detail using molecular dynamics simulations although with such a large ensemble (28,847 atoms plus water) only short simulation times (totaling < 10 ns) have been so far achieved by this group (Fig. 16). Experience with atomistic simulations of the troponin core indicate that a steady state is reached only after about 100 ns and that the disordered parts of the structure do not reach convergence on any structure, even after 10 s of µs, indicating this structure is provisional (Zamora et al. 2016; Zamora 2019).

Gould and Zamora have developed a smaller model system (15 vs. 25 actins long) with restoration of native sequences, separate + and − Ca^2+^ simulations and different computational methods to obtain simulations of at least a microsecond which has the potential for providing an analytical solution to troponin regulatory structure and dynamics changes in the thin filament comparable with that already done with troponin (Zamora 2019).

## How the Ca^2+^ switch works at the atomic level

The mechanism by which troponin switches the activity of the thin filament in response to Ca^2+^ has been established for some time. At low cytosolic Ca^2+^ levels, the formation of the actomyosin complex is sterically inhibited by Troponin I (TnI), through its C terminus binding to actin and locking tropomyosin in a blocking position such that strong binding of actin to myosin is not allowed. When the levels of Ca^2+^ increase, a single Ca^2+^ ion binds to the regulatory N-terminal cTnC site II. It provokes an intra-molecular conformational shift, opening up a hydrophobic patch of NcTnC and exposing it for interaction with the cTnI switch peptide, which is also associated with a hinge motion of the N-terminal TnC domain relative to the IT domain (Figs. 5, 8, 14). Binding of the TnI switch peptide to the hydrophobic patch, in turn, pulls the C terminus of TnI away from actin. This permits a cooperative shift of the tropomyosin molecule across the actin surface, exposing all the myosin binding sites on actin and thus permitting cross-bridge cycling.

**Fig. 17:**
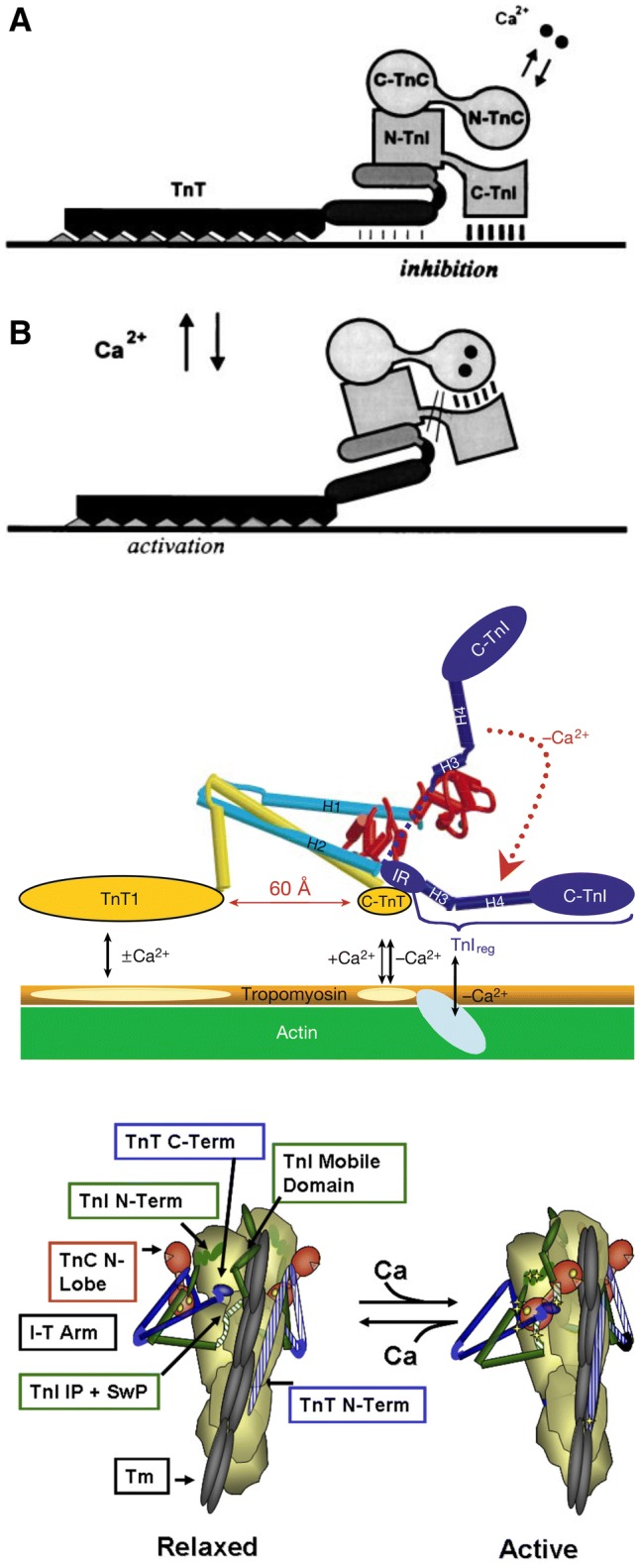
Structural models describing Ca^2+^ regulation of thin filaments by troponin

This has been incorporated into structural models, three of which are shown in Fig. 17; the fundamental mechanism has not changed, but with each iteration more detail has been added. The model by Malnic et al. (1998) shows the state of the art achieved by site-directed mutagenesis studies on the troponin complex. The fundamental Ca^2+^ switching is achieved by the switch of TnI C terminus binding from actin, where it inhibits, to the troponin C hydrophobic patch when Ca^2+^ is bound.

The model of (Takeda et al. 2003) incorporates this into the 3D model of troponin obtained by X-ray diffraction; with the precise interacting sequences identified.

The final model from Solaro and Kobayashi (2011) now incorporates the whole thin filament into the scheme; in this case the known interactions between the regulatory proteins are all accounted for although the structural arrangements remain speculative.

**Fig. 18:**
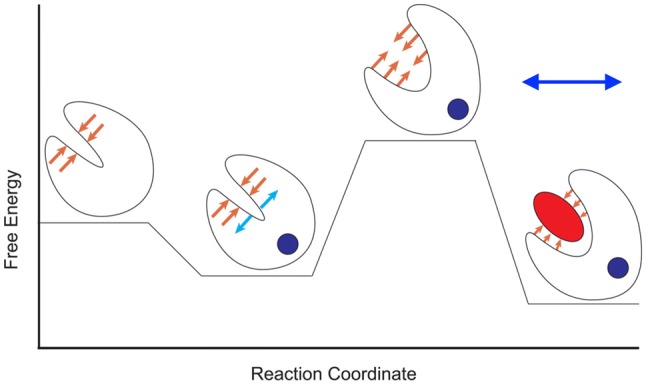
Schematic of the energetic landscape of N-cTnC activation. N-cTnC is shown as a cartoon. Ca^2+^ is a blue circle, and the TnI switch peptide is represented as a red ellipse. Lower energy states are more favourable. The orange arrows represent the resistance to the conformational change caused by the hydrophobic cleft. The blue arrows indicate conformational strain introduced by Ca^2+^ binding. The Ca^2+^-bound, open conformation relieves the conformational strain while occluding the hydrophobic cleft and is therefore the most favourable conformation. From (Stevens et al. 2017), with permission. The line represents the range of the system accessed by phosphorylation and mutations. (Color figure online)

Models like these are based on fixed structures and do not represent the dynamic nature of the thin filament. This is an area that has begun to be tackled. Lehman and Orzechowsky have considered the regulatory conformational changes of the ‘steric blocking’ mechanism of actin-tropomyosin in terms of an energy landscape in which tropomyosin is located in a set of energy wells on the actin surface whose relative stability and energy barriers between states is dictated by the regulatory state (open, closed and blocked) controlled by troponin, Ca^2+^ and myosin heads (Kiani et al. 2018; Orzechowski et al. 2014; Lehman 2017). Likewise, the troponin Ca^2+^-switch may be represented as a reaction coordinate where the probability and rates of progress between multiple states from inactive to active is determined by the Ca^2+^ binding and the associated energy barriers (Dong et al. 1999; Stevens et al. 2017) (Fig. 18).

It is noteworthy that mutations and post-translational modifications have rather small effects on Ca^2+^-sensitivity and generally do not change average structure, but rather alter the dynamics of the Ca^2+^ switch resulting in a change in Ca^2+^-sensitivity. (Dong et al. 2008; Marston et al. 2013; Stevens et al. 2017).

## Modulation of the Ca^2+^ switch by troponin I phosphorylation

**Fig. 19:**
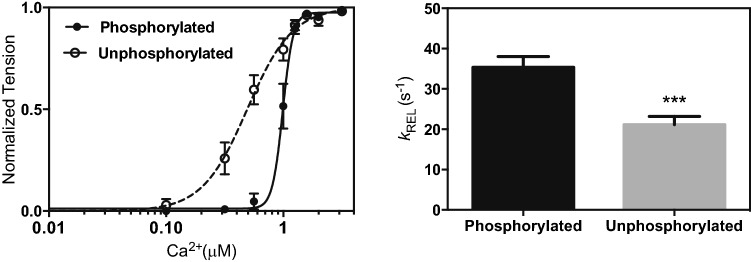
Effect of dephosphorylation on mouse myofibril contractility. **a** The Ca^2+^-sensitivity curve of isometric force for unphosphorylated myofibrils (open circles, dashed line) is shifted to the left of the phosphorylated myofibrils (solid circles, solid line. **b** Kinetic parameter k_REL_ at maximally activating Ca^2+^ and SL 2.17 µm. From Vikhorev et al. (2014)

The heart possesses a unique regulation mechanism that allows it to meet increased oxygen demand from the rest of the body. During periods of high stress or increased physical activity, catecholamines such as adrenaline and noradrenaline are released into the bloodstream and from sypathetic nerve endings and subsequently bind to *β*-1 receptors in cardiac myocytes. This leads to activation of adenyl cyclase via Gs, an increase of the cytosolic cyclic adenosine monophosphate (cAMP) levels and activation of protein kinase A (PKA), which phosphorylates cTnI and other sarcomeric proteins. cTnI is phosphorylated at residues Ser22 and Ser23 in the N-terminal peptide (residues 1 to 36), which is exclusive to the cTn isoform (Al-Hillawi et al. 1995; Ayaz-Guner et al. 2009; Mittmann et al. 1990). The effect of this post-translational modification is a decrease of affinity of cTnC for Ca^2+^ due to altered cTnC-cTnI interactions linked with a higher rate of Ca^2+^ dissociation from cTnC (Dong et al. 2007; Robertson et al. 1982; Solaro et al. 1976). See Fig. 19.

The faster relaxation rate (lusitropy) of the cardiac muscle due to TnI phosphorylation is essential for shortening the heart contraction-relaxation cycle allowing for a faster heart rate.

Although the physiological implications of the modulation of the Ca^2+^ switch by TnI phosphorylation are well understood, the underlying structural mechanism by which it occurs has remained elusive. The interaction of the N-terminal phosphorylatable peptide of TnI with TnC has been the centre of attention for studies on this process.

Phosphorylation of cTnI has negligible effects on the N-domain conformation but it affects Ca^2+^ sensitivity and modifies the kinetics of opening and closing of the N-domain induced by binding of Ca^2+^ and S1 (Dong et al. 2007). A seminal NMR study by Baryshnikova, M. X. Li, and Sykes (Baryshnikova et al. 2008a) on partial fragments of the core region of cTn established that both the N-terminus of TnI (1-33, NcTnI) and the switch peptide simultaneously bind to the N-terminal domain of TnC (NcTnC) (see Table 2). The switch peptide was found to bind to NcTnC in the same region regardless of the presence of NcTnI in both unphosphorylated (uP) and phosphorylated (P) states, but in the absence of NcTnI this interaction is tighter (*K*_*d*_= 154 μM). Additionally, the binding of NcTnI to NcTnC was found to be more efficient in the uP state, and that phosphorylation created no new conformational changes to NcTnC. Interestingly, this study reported that the phosphorylation signal provokes an increased strength of interaction between the cTnI switch peptide and NcTnC (in the P state, *K*_*d*_= 370 ± 30 μM compared to *K*_*d*_= 600 ± 100 μM in the uP state) rather than by a direct alteration of the Ca^2+^ interaction with cTnC in its binding pocket, EF-hand II (*K*_*dCa*_= 5 ± 3 μM in both the P and uP states). This two-fold difference between uP and P cTn in the switch peptide—NcTnC affinity of interaction is comparable with experiments in whole thin filaments and can account for the differences in relaxation rates of the thin filament. In fact, Baryshnikova et al. point out that the ordering of cTnI’s switch peptide—NcTnC strength of interactions match with the inverse order of the relaxation speed of myofibrils under different physiological conditions: no NcTnI > NcTnI P > NcTnI uP.

This behaviour is comparable with other studies on enzyme regulation by phosphorylation where several unifying themes have been proposed (Colson et al. 2012). Firstly, the effect of phosphorylation changes the structure of the bound complex without dissociation, secondly that phosphorylation induces transitions between order and dynamic disorder (or changes in the magnitude of disorder), as demonstrated by Hwang for TnI-TnC (Hwang et al. 2014) and, finally, structural states are only loosely coupled to phosphorylation; i.e., complete phosphorylation induces dramatic functional effects with only a limited shift in the equilibrium between ordered and disordered. In terms of the troponin Ca^2+^ switch, the phosphorylation effects are limited to affecting the kinetics and equilibrium of the TnI/Ca^2+^/open (active) configuration of troponin C (right hand end of Fig. 18) that is separated from the rest of the activation pathway by a large energy barrier.

Recently computational molecular modelling and molecular dynamics simulations have become the method of choice for understanding this system. The first MD study of the structural effects of cTnI phosphorylation was performed by (Cheng et al. 2014, 2015). This study used a reasonably complete structure of the core domain of cTn: the complete sequence of cTnC (residues 1 to 161), cTnI (residues 1 to 172) and cTnT (residues 236 to 285). The authors compared unphosphorylated cTn to pseudophosphorylated cTn using a double phosphomimic substitution: cTnI S23D/S24D. The main observations were the formation of a new interaction between NcTnI and the ‘inhibitory’ peptide after pseudo- phosphorylation and an overall increase in the system’s fluctuation profiles. This observation was similar to an NMR study on the conformational effects of cTnI phosphorylation by Howarth et al. (2007). It was proposed that phosphorylation caused an extension of a helical motif at the end of the NcTnI region which reduced the interactions between NcTnC and NcTnI. Thus, the first few acidic residues of NcTnI were positioned to interact with the positively-charged ‘inhibitory’ peptide of cTnI, defining a new and potentially significant interaction in troponin.

A more extensive study by Zamora et al. (2016) replicated the simulations of Cheng et al., using a complete model of the core domain of cTn, an updated force-field and substantially more sampling (both a larger volume simulated and 10 × longer times). This study failed to reproduce the reported effects of phosphorylation on the structure and dynamics of cTn. It was found that the interactions between the NcTnI region and the inhibitory peptide were only occasionally sampled in any phosphorylation state and that there was a lack of convergence between independent simulations despite the longer simulation times. This suggests the results in the original publication could have been random events. Although some apparent phosphorylation-related changes were observed, Zamora et al’s main conclusions were that the structural and dynamic changes induced by phosphorylation could not be defined with any statistical significance using only a small number of independent MD trajectories a few hundred ns of length.

**Fig. 20:**
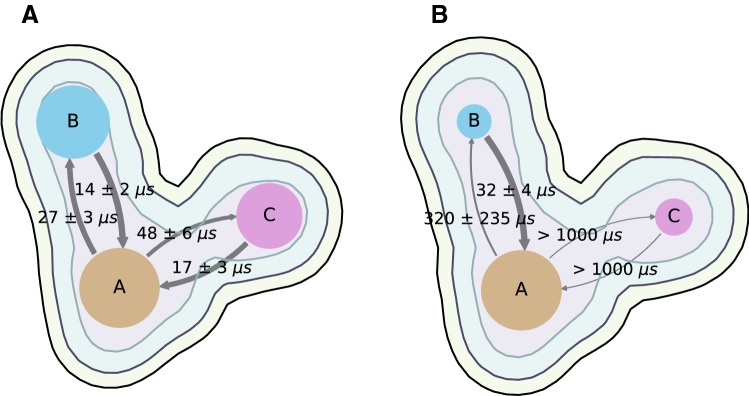
The three macrostates of troponin located in their average positions on the landscape formed by the first two time-structure Independent Components Analysis. **a** WT unphosphorylated macrostates and the MFPTs of transition between them. **b** WT Phosphorylated macrostates and the MFPTs of transition between them. The node sizes reflect the relative stability of the macrostates, the arrow labels indicate the Mean First Passage Times of transition in μs (Zamora 2019)

Recently Zamora et al. have produced and analysed a substantially larger dataset (over 30 µs simulation of both P and unP troponin) produced with an updated force field (Zamora 2019). This new force field, termed ff14SB, belongs to the AMBER protein force fields and represents a revised and updated version of ff99SB (Maier et al. 2015). A complete refit of the side chain dihedral parameters was performed. Overall, this improved secondary structure predictions and NMR scalar couplings for proteins in solution. Additionally, they used Markov State Models (MSMs) to understand the dynamic transitions between the structural ensembles of cTn. This revealed a significant difference between phosphorylated and unphosphorylated troponin (Fig. 20).

MSMs are a suitable technique to analyse MD simulations since they model the transitions between a set of disjoint states as a memoryless network. The mathematical underpinnings of this analysis technique have been known for a long time (Husic and Pande 2018). However, their applicability to the study of complex biomolecular processes has experienced a surge of popularity thanks to improvements in the estimation of these models and in the increased capabilities of generation of simulated dynamical data into timescales of biological relevance (Prinz et al. 2011). MSMs require the partition of the structural phase space into hundreds, or even thousands, of microstates to provide a highly detailed description of the conformational dynamics in multidimensional space. The resulting models are thus very complex: to aid in their interpretability, it is often desirable to derive from them simple kinetic models with la ow number of states. The objective is to obtain a handful of metastable macrostates formed by MSM microstates which interconvert quickly between them. Based on the spectral gap that is observed in the timescales, Zamora et al. decided to partition of the space into three macrostates. The relative population of the macrostates at equilibrium and their interconversion rates are altered upon phosphorylation.

The simulation data may also be interpreted in terms of the current models for the Ca^2+^-switch. Zamora et al. followed the model of Kekenes-Huskey et al. (2012) (Fig. 18). Because the simulations are in the Ca^2+^-activated state, the cTnI switch peptide is always bound to the hydrophobic patch and was never observed to unbind in any of the simulations.

The cTnC helix A–B interhelical angle was calculated as a function of time. Both unphosphorylated and the phosphorylated states remained largely in the open conformation, but, transitions to the closed conformation were observed to be more frequent in the WT apo unphosphorylated state. From time-series data, it was possible to estimate the free-energy barrier associated to this conformational rearrangement, as well as the mean first passage time (MFPT) to go from an open to closed conformation. It was observed that the energy barrier associated for the open-to-close conformation of the hydrophobic patch of cTnC is lower for the unphosphorylated state than for the phosphorylated state, with a ∆∆*G*_*uP*→*P*_≈ 27 kcal mol^−1^. Furthermore, the maximal angle that is observed for the unphosphorylated state is 141°, whilst the maximal angles that are achieved in the phosphorylated simulations is 119°. Finally, the kinetics of closing are also altered, since the MFPT between the closed and open states is on the low microsecond time regime in the unphosphorylated state, whilst it is increased by an estimated order of magnitude in the phosphorylated state, suggesting phosphorylation enhances the probability of the open state.

**Fig. 21:**
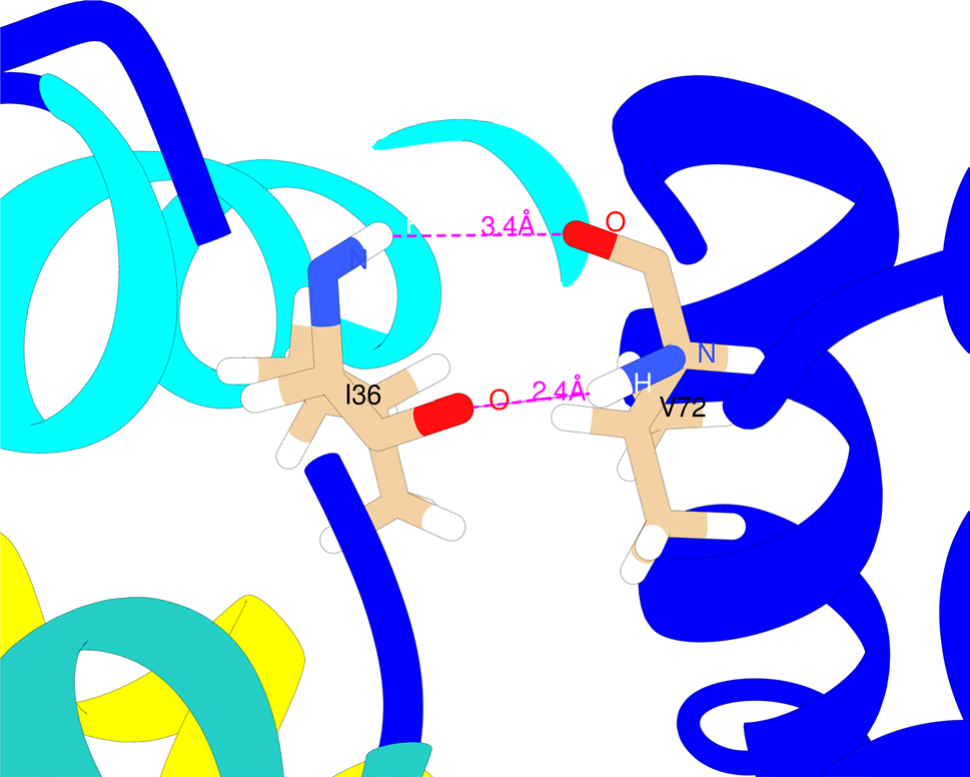
The two backbone hydrogen bonds that can be formed between cTnC I36 and V72 in the open state and that are modulated by phosphorylation

In addition helicity was investigated throughout the troponin sequence. The average change in the propensity to form a randomly coiled structure was mapped onto each residue of the cTn core domain. As can be appreciated, a large portion of cTn is unaltered upon phosphorylation. Of the residues that are changed, the I36-V72 pair were of particular interest since they are not sequential but they are spatially close. I36 and V72 are located in the loops connecting helices A–B and C–D, respectively, and can interact through backbone hydrogen bonds in the open state (see Figs. 5, 6 and 21). Thus, it was hypothesised that the increased frequency of isolated *β* bridge formation between I36 and V72 could be the reason behind the lower frequency of conformational change that is observed in the NcTnC hydrophobic path in the phosphorylated states. Additionally, V28, L29 and G40 are located at the end of helix A: their propensity towards disorder in the phosphorylated state could also contribute to this mechanism—a less rigid helix could have a harder time pivoting towards the closed conformation of the hydrophobic patch. It was calculated that phosphorylation triggers an increase of about 10% in the cluster population corresponding to having both hydrogen bonds present. Interestingly, not only are the relative populations of the hydrogen bonds altered by phosphorylation, but also the timescales associated with the creation and dissociation of the bonds. Overall these simulations indicate that phosphorylation stabilises the open state, in a similar way to that proposed by Barashnikov et al. (2008a).

The model derived from molecular dynamics offers an explanation as to how mutations could uncouple TnI phosphorylation from the change in Ca^2+^-sensitivity, a phenomenon seen with many HCM and DCM-related mutations (Memo et al. 2013; Messer et al. 2016; 2017). Early studies indicate that mutations destabilise the open state (Zamora 2019).

## Modulation of the Ca^2+^ switch by mutations

**Fig. 22:**
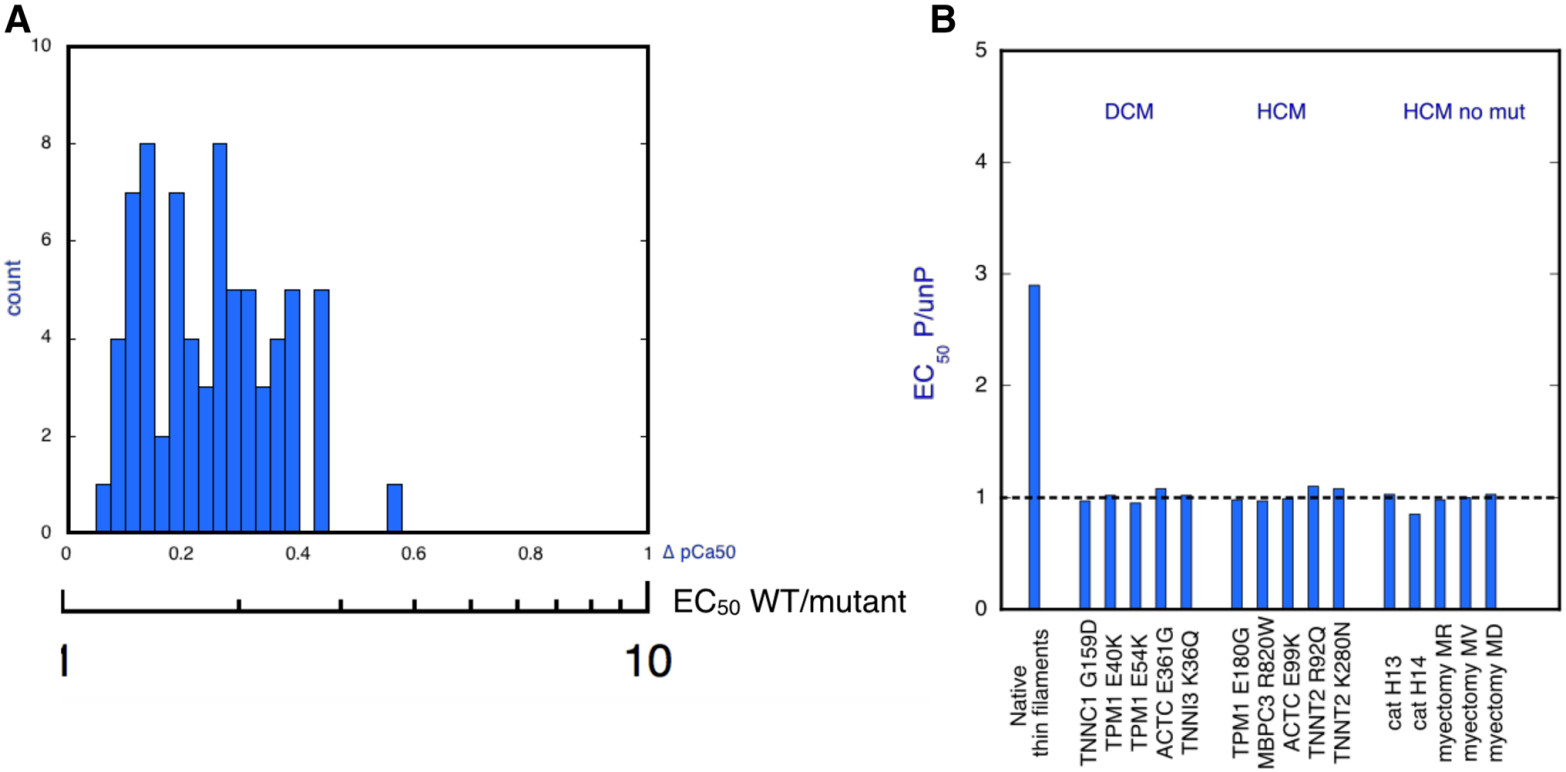
**a** Frequency histogram of increase in Ca^2+^ sensitivity for 71 measurements of HCM mutations compared with wild-type. The plot includes results obtained with 4 measurement methods with 44 mutations in 6 genes. The mean increase in Ca^2+^ -sensitivity is 1.87 ± 0.07 fold (sem). From (Marston 2016). **b** Uncoupling of the relationship between cTnI phosphorylation and myofilament Ca^2+^ -sensitivity. The ratio of EC_50_ phosphorylated: Unphosphorylated is 2.4. In wild-type, but 1.0 for the HCM and DCM mutations shown on the x-axis, indicating uncoupling. From (Sheehan et al. 2018)

A large number of mutations in troponin subunits have been found to be associated with cardiomyopathies and have been extensively studied. Mutations in TNNNT2 and TNNI3 genes are significant causes of hypertrophic cardiomyopathy and dilated cardiomyopathy, whilst mutations in in TNNC1 are rare causes of these cardiomyopathies, Functionally the HCM mutations all seem to increase myofilament Ca^2+−^sensitivity by about twofold (Marston 2011, 2016) and this is proposed to be disease-causing (Spudich 2014). In contrast, the effect of DCM mutations on Ca^2+^ sensitivity is variable (Marston 2011; Memo et al. 2013). In addition, in many HCM mutations and all DCM mutations in troponin subunits so far tested the relationship between TnI phosphorylation by PKA and decreased Ca^2+-^sensitivity is abolished (Dvornikov et al. 2016; Dyer et al. 2009; Messer et al. 2016; Messer and Marston 2014) and this appears to be sufficient to cause DCM in the long term (Fig. 22) (Wilkinson et al. 2015).

Only a few mutations have been studied at the structural level; as would be expected from their small functional effects, the structural effects of mutations are subtle.

*The TnI mutation R145W* has been extensively studied since it was reported to be associated with particularly severe HCM or restrictive cardiomyopathy and because it is located in the ‘inhibitory’ peptide of TnI (Deng et al. 2001; Elliott et al. 2000; Kruger et al. 2005; Lang et al. 2002). It has been established that this mutation increases Ca^2+^-sensitivity and uncouples TnI phosphorylation from a Ca^2+^ sensitivity change (Dvornikov et al. 2016). Two molecular dynamics studies of the troponin core have addressed this mutation. The study of Lindert et al. (2015) may have been compromised by the size of the box used for simulation (Papadaki and Marston 2016; Zamora et al. 2016). However, a study by Dvornikov et al. (2016) used a larger box, although simulation times were still short (100 ns). The mutation did not have any global effects but it was found to cause a significant local reduction in the interaction of amino acid 145 with acidic residues in the cTnC C helix (E63, E59, E56) which was not phosphorylation-dependent. How the mutation affects dynamics and the mechanism for increased Ca^2+^-sensitivity was not apparent from this study.

*The L29Q mutation in cTnC* has also been investigated. This mutation is regarded as an HCM-causing mutation although the clinical and genetic data is not very extensive (Hoffmann et al. 2001). It has been studied by X-ray diffraction, NMR and fluorescence polarisation (Li et al. 2013; Robertson et al. 2015; Zhang et al. 2013). All these methodologies indicate that the mutation did not perturb the overall structure although some local changes were observed.

*The troponin C G159D mutation* is associated with a severe dilated cardiomyopathy and is one of the most thoroughly investigated DCM mutations (Biesiadecki et al. 2007; Dyer et al. 2009; Kaski et al. 2007).

An NMR spectroscopy study by Baryshnikova et al. (2008b) found little difference in structure or dynamics of Mutant TnC, C terminal domain interacting with TnI 34-71 although the affinity of this interaction was reduced from < 1 µM in wild-type to 3.0 µM in G159D. The significance of this is hard to assess since the structures studied were only fragments of troponin and did not include the regulatory head of troponin or TnT.

Zamora and Gould have analysed the G159D mutation in their model of the complete troponin core in both phosphorylated and unphosphorylated states. Remarkably, simple RMSF plots indicate that phosphorylated G159D is significantly less mobile (a generalised reduction in all three subunits) compared with unphosphorylated G159D and wild-type, phosphorylated or unphosphorylated, which were all similar. This indicates that Phosphorylated G159D is uniquely more rigid. This is reflected in calculations of the helix A/B angle where a significant population of more closed angles was observed in phosphorylated G159D, opposite to the effect of phosphorylation on wild-type. This suggests a decreased probability of the open state and may account for the lack of a decrease in Ca^2+^-sensitivity coupled to phosphorylation in G159D (Zamora 2019).

Kekenes-Huskey et al. examined a number of mutations that affect Ca^2+^ affinity in a model of NcTnC and noted that helicity and/or the A/B helix angle dynamics could be altered (Kekenes-Huskey et al. 2012). Recently Stevens et al. conducted an extensive molecular dynamics study of 6 HCM -linked mutations in the N terminal domain of TnC including L29Q (Stevens et al. 2017). The mutations modified the structural dynamics of TnC rather than the regulatory Ca^2+^-binding site. The changes are observed in the relative favourability of the protein conformations that transduce the contraction signal by destabilizing the closed conformation of N-cTnC, stabilizing the open conformation, or stabilizing the interaction of NcTnC with the TnI switch peptide.

In summary, the effects of mutations on troponin seem to mimic the effect of phosphorylation/dephosphorylation in that they modulate the dynamics of the Ca^2+^ switch elements (Fig. 18). This rather general mechanism may explain how mutations in many different proteins of the sarcomere can produce the same phenotype at the molecular level.

## Conclusions

Recent investigations of troponin have confirmed the basic mechanism of the Ca^2+^-switch, established over the last 20 years and have added structural detail. Present studies are mainly directed towards the subtle modulation of the switch by phosphorylation and mutations. These do not produce detectable changes in the steady state but in both cases significant changes in troponin dynamics, exemplified by the A/B helix angle, can be demonstrated by NMR and molecular dynamics simulations. Future studies on troponin structure and function will need to address in detail the intrinsically disordered parts of troponin that play an essential role in both the Ca^2+^ switch and its physiological and pathological modulation.
